# University of Buffalo

**Published:** 1905-10

**Authors:** Edwin Fleming, Frank H. Severance, F. Hyatt Smith, Edward D. Strickland, Charles E. Norton

**Affiliations:** Publicity Committee, 117 Erie County Bank Building, Buffalo, N.Y.; Publicity Committee, 117 Erie County Bank Building, Buffalo, N.Y.; Publicity Committee, 117 Erie County Bank Building, Buffalo, N.Y.; Publicity Committee, 117 Erie County Bank Building, Buffalo, N.Y.; Chairman, 17 Erie County Bank Building, Buffalo, N.Y.


### University of Buffalo,

**University Extension Movement.**

*Editor Buffalo Medical Journal:*

Sir:—We are now busily engaged in the work of organising and maturing the movement to create a college of letters and of sciences as a department of the University of Buffalo, of which so frequent mention has been made in the Buffalo newspapers during the past few months. The movement is to be carried on by addresses made to the various associations of the City of Buffalo, and by the circulation of subscription papers throughout the entire city and the towns in the immediate vicinity of Buffalo which would naturally furnish students to such a college when once organised. The main object of the movement is to reach, as far as possible, every man and woman in the city and the vicinity, and to appeal to them to take some part in the movement to give to the City of Buffalo and vicinity a municipal college such as other cities of its size already have, thereby enabling the bright young men and women of the city and vicinity to obtain a college education, where, otherwise, many of them will be unable to do so as they cannot afford to go away from home to get it. It is of the greatest importance, of course, to secure the aid of the entire press in this movement, for without such aid success is problematical, but with it we feel satisfied that success is assured. This circular letter, sent to all papers in the territory likely to furnish students or to be benefited by such a college, is to ask their assistance and continued co-operation in this important movement. We would suggest the benefits likely to accrue, space permitting, by presenting in your columns as much as possible of what appears in those of your exchanges, thereby unifying the effort and giving it greater force. And it would be a further favor, and one which will be much appreciated, if you will have marked copies, containing matter pertinent to the movement, mailed to Charles P. Norton, 117 Erie County Bank Building.

Edwin Fleming,
Frank H. Severance,
F. Hyatt Smith,
Edward D. Strickland,
*Publicity Committee.*
Charles E. Norton, *Chairman.*

117 Erie County Bank Building, Buffalo, N.Y., September 1, 1905.

